# Less Is More? Two Cases of Cryptococcosis Treated Using Single-dose Liposomal Amphotericin B as Part of Induction Therapy in Solid Organ Transplant Recipients

**DOI:** 10.1097/TXD.0000000000001648

**Published:** 2024-05-28

**Authors:** Carson K.L. Lo, Christie Rampersad, Justin Barr, Shahid Husain

**Affiliations:** 1 Division of Infectious Diseases, Department of Medicine, McMaster University, Hamilton, ON, Canada.; 2 Transplant Infectious Diseases and Ajmera Transplant Centre, University Health Network, University of Toronto, Toronto, ON, Canada.; 3 Transplant Nephrology and Ajmera Transplant Centre, University Health Network, University of Toronto, Toronto, ON, Canada.; 4 Institute of Health Policy, Management and Evaluation (IHPME), Dalla Lana School of Public Health, University of Toronto, Toronto, ON, Canada.; 5 Department of Abdominal Transplant Surgery, Ajmera Transplant Centre, University Health Network, University of Toronto, Toronto, ON, Canada.

In solid organ transplant (SOT), cryptococcosis causes invasive fungal infection by reactivated latent infection (median onset 15–19 wk posttransplantation), donor-derived transmission, or posttransplant de novo infection.^1,2^ Clinical presentations typically include pulmonary, central nervous system (CNS), and less commonly skin, bone, and rarely prostate or bladder.^2,3^ Overall, 30-d mortality approaches 20% despite treatment.^1,4^ Treatment regimens are also commonly associated with adverse effects, highlighting a need for refined strategies.^5^

The AMBITION trial was a phase 3 noninferiority randomized controlled trial among 844 patients with HIV and cryptococcal meningitis comparing 2 induction protocols: (1) single high-dose liposomal amphotericin B (l-AmB, 10 mg/kg), 2-wk flucytosine (100 mg/kg/d), and fluconazole (1200 mg/d), versus (2) World Health Organization–recommended 1-wk amphotericin B deoxycholate (AmBd, 1 mg/kg/d) and flucytosine (100 mg/kg/d) followed by 1-wk fluconazole (1200 mg/d). The single-dose l-AmB protocol was noninferior for all-cause mortality at 10 wk, achieved similar fungal clearance, and reported fewer adverse events.^6^ World Health Organization guidelines subsequently adopted the AMBITION regimen for treating cryptococcal meningitis in people living with HIV (PLWH).^7^

Although largely extrapolated from studies in PLWH, current SOT guidelines recommending 2-wk minimum l-AmB 3–4 mg/kg/d combined with flucytosine 100 mg/kg/d predated the AMBITION trial.^2^ Although no SOT recipients were included in the trial, the AMBITION regimen is of interest in SOT because of shorter duration requiring intravenous administrations, reduced toxicity profile, and less monitoring for adverse events.^6^ The 2024 joint guidelines by the European Confederation of Medical Mycology, the International Society for Human and Animal Mycology, and the American Society for Microbiology (ECMM/ISHAM/ASM) highlighted that evidence for the AMBITION regimen with high-dose fluconazole and its ensuing potential drug-drug interactions and toxicity is absent in SOT recipients.^8^ In the absence of clinical trials, real-world accounts in SOT recipients are needed. We present the first 2 reported cases of cryptococcosis in SOT recipients successfully treated with the AMBITION regimen.

## CASE DESCRIPTION

### Case 1

**FIGURE 1.**
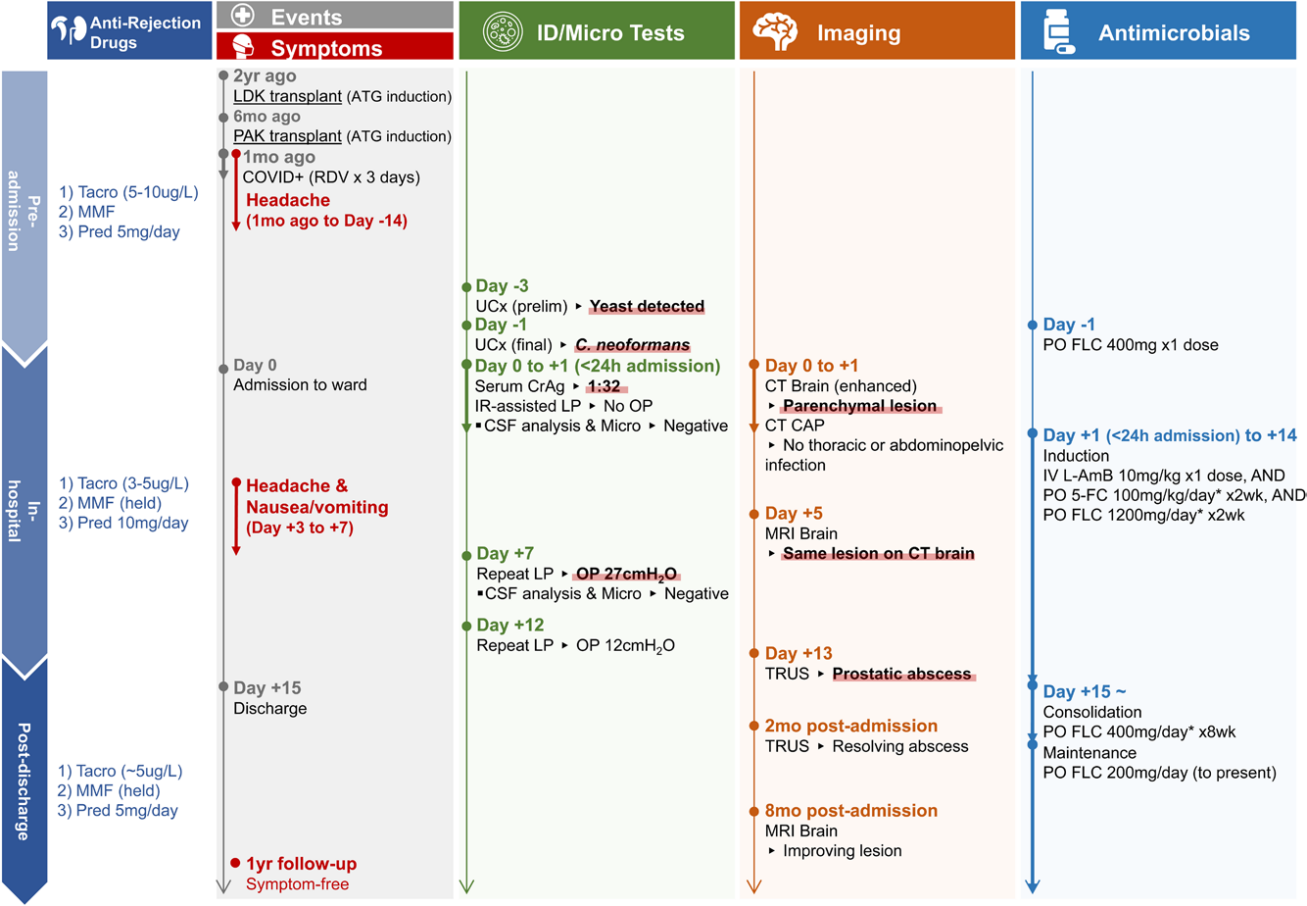
Clinical presentation timeline of case 1. Day 0 represents the day of hospital admission. The vertical timeline axes are measured at nonequidistant intervals. *Medications requiring renal dose adjustment. 5-FC, flucytosine; ATG, antithymocyte globulin; CAP, chest-abdomen-pelvis; CrAg, cryptococcal antigen; CSF, cerebrospinal fluid; CT, computed tomography; FLC, fluconazole; ID, infectious diseases; IR, interventional radiology; IV, intravenous; LDK, living donor kidney (transplant); LP, lumbar puncture; micro, microbiology; OP, opening pressure; PAK, pancreas-after-kidney; PO, per os; pred, prednisone; prelim, preliminary; RDV, remdesivir; tacro, tacrolimus (with target dose range in µg/L); TRUS, transrectal ultrasound; UCx, urine culture.

A 49-y-old man with kidney failure attributed to type 1 diabetes underwent a preemptive living donor kidney transplant and pancreas-after-kidney transplant 1.5 y later (Figure 1). Immunosuppression was antithymocyte globulin induction for both transplants and maintenance tacrolimus (target trough 5–10 µg/L), mycophenolate mofetil (MMF), and prednisone (5 mg/d).

He presented 6-mo post-PAK transplant with headaches and nonproductive cough; COVID-19 was diagnosed and treated with clinical improvement. One month later, he developed cloudy urine. An outpatient urine culture revealed *Cryptococcus neoformans* and he received a single dose of 400 mg of oral fluconazole.

On hospital ward admission, the assessment noted cloudy urine, anorexia, and hypovolemia. Bloodwork showed normal white blood cell (WBC) count, lymphopenia (0.3 × 10^9^/L), and serum creatinine 170 µmol/L (baseline 120 µmol/L). Intravenous fluids were initiated, MMF was discontinued, and prednisone increased (10 mg/d).

**FIGURE 2.**
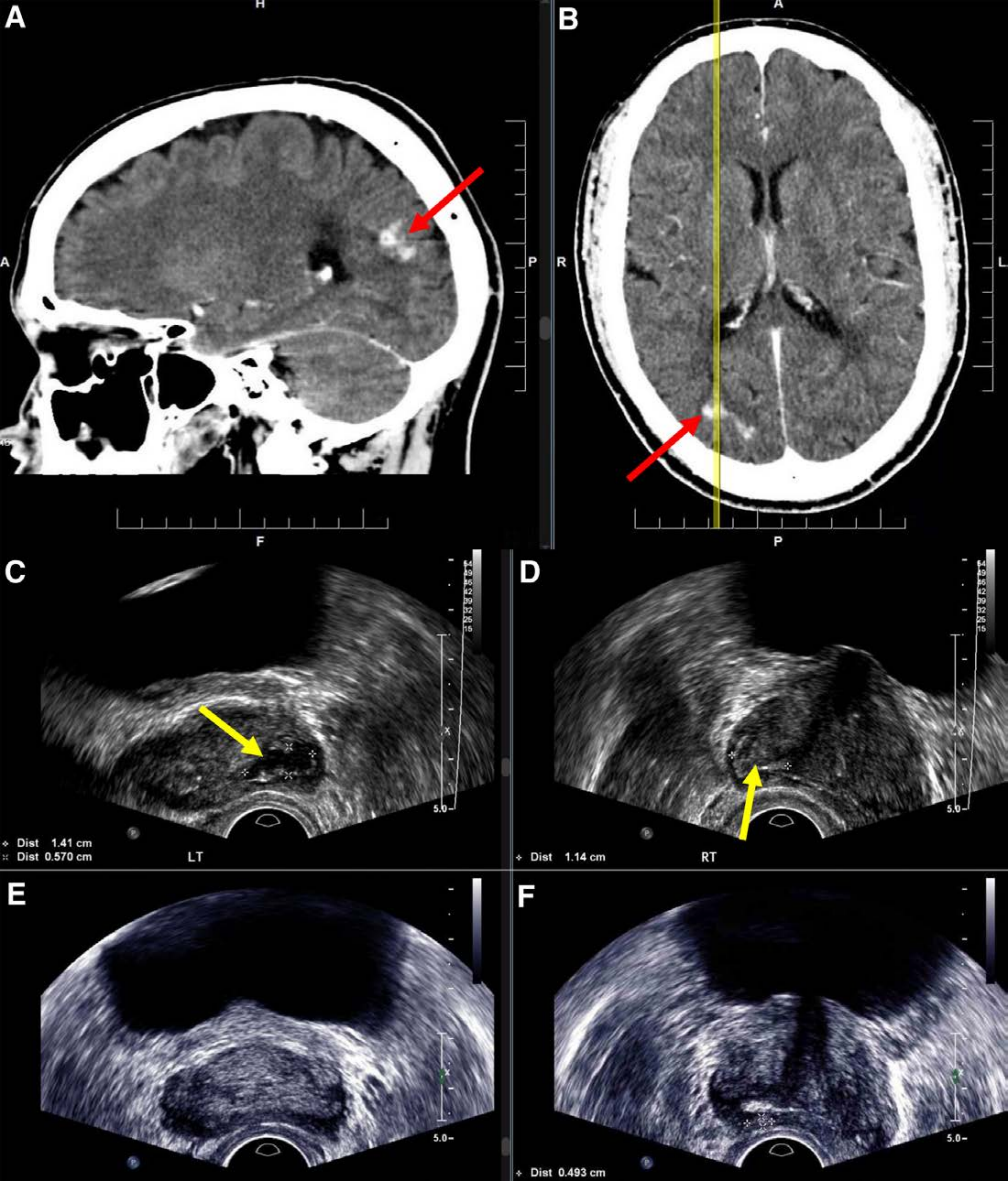
Imaging of case presentation. Contrast-enhanced computed tomography with select sagittal (A) and axial (B) images of the brain (yellow line indicates corresponding sagittal plane), showing enhancing parenchymal lesion (red arrow) with multifocal nodules near the right lateral ventricular horn. Transrectal ultrasound of prostate (C–F). Within 2 wk of diagnosis, left (C) and right (D) prostate bases showing fluid collections (yellow arrow). Two months later, similar imaging views of the prostate base showing resolution of the left-sided (E) and improving right-sided (F) collection.

Serum cryptococcal antigen (CrAg) was positive (titer 1:32). Contrast-enhanced computed tomography (CT) head demonstrated enhancing parenchymal lesion suspicious for cryptococcoma (Figure 2). A CT scan of the chest-abdomen-pelvis demonstrated no metastatic disease.

He was treated as presumed disseminated cryptococcosis with possible CNS involvement using the AMBITION induction protocol (single-dose l-AmB (10 mg/kg), 2-wk flucytosine (100 mg/kg/d), and fluconazole (1200 mg/d), dose adjusted for kidney function).^6^ Acute kidney injury (AKI) was attributed to pre–renal hypovolemia and acute tubular necrosis from l-AmB. Creatinine peaked at 264 µmol/L before steadily improving.

Before induction, lumbar puncture (LP) yielded clear and colorless cerebrospinal fluid (CSF). Opening pressure was unmeasured. CSF analysis showed WBC count 1 × 10^6^/L, glucose 4.2 mmol/L, protein 0.6 g/L, negative CrAg by latex agglutination, and negative bacterial and fungal culture, and polymerase chain reaction (PCR) by BioFire FilmArray Meningitis/Encephalitis panel.

Brain MRI redemonstrated CT findings. Repeat LPs performed for new headache and intractable nausea had maximum opening pressure of 27 cmH_2_O; opening pressure and symptoms improved with serial LPs. Repeat CSF analysis remained unchanged and negative.

Transrectal ultrasound of the prostate (Figure 2) revealed a fluid pocket at the left (1.1 × 1.4 × 0.6 cm) and heterogenous echogenicity at the right (1.1 × 0.8 × 0.5 cm) base. Although suspicious for fungal abscesses, they were too small for aspiration.

Following a 2-wk induction protocol, he was discharged home with 8-wk consolidation, then planned 1-y maintenance with fluconazole monotherapy. Repeat transrectal ultrasound 2 mo and brain MRI 8 mo postinduction demonstrated improving lesions. At the 1-y follow-up, he remained symptom free with stable kidney-pancreas allograft function on dual immunosuppression (tacrolimus/prednisone) and remains off MMF indefinitely.

### Case 2

**FIGURE 3.**
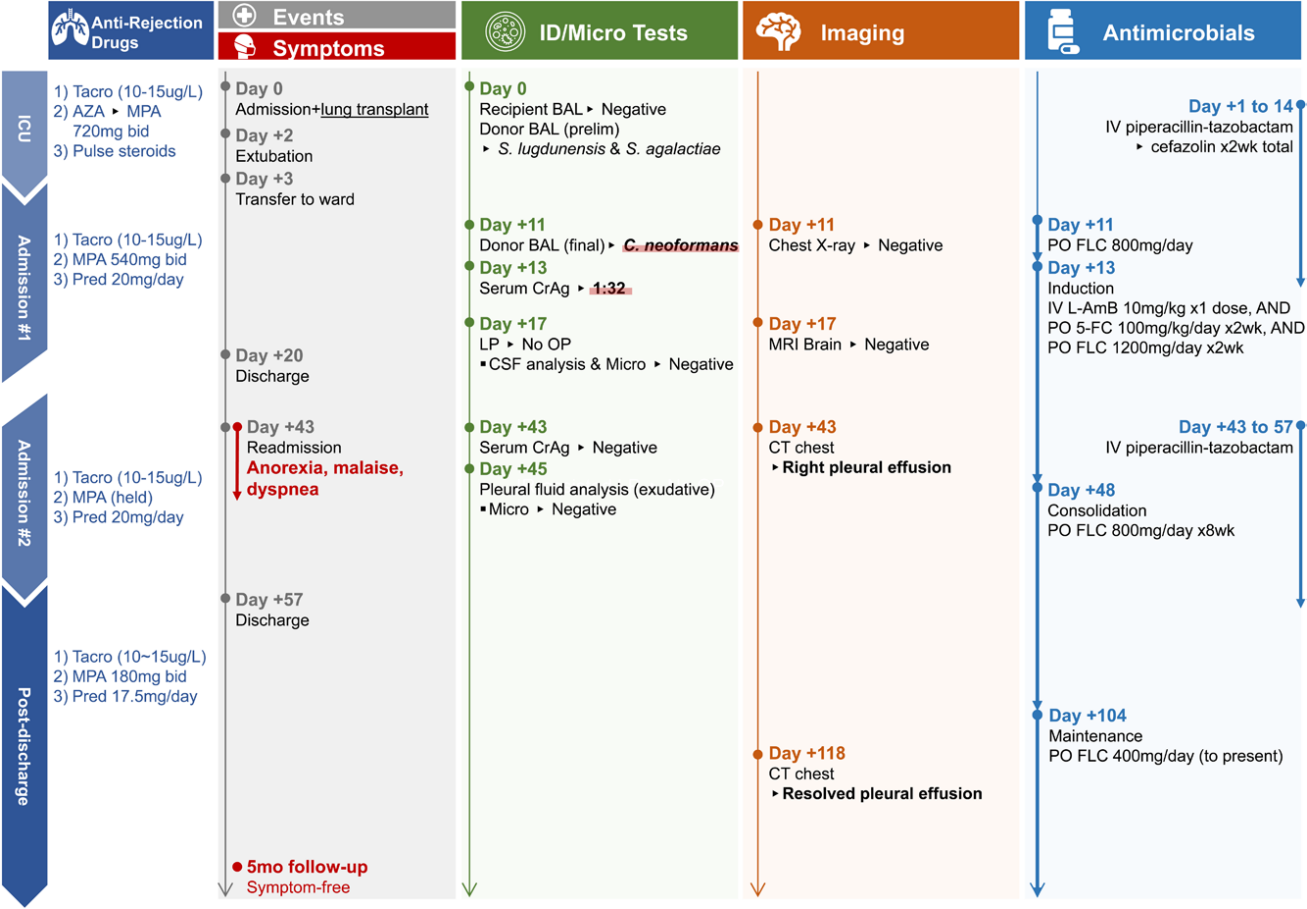
Clinical presentation timeline of case 2. Day 0 represents the day of hospital admission and lung transplantation. The vertical timeline axes are measured at nonequidistant intervals. 5-FC, flucytosine; AZA, azathioprine; BAL, bronchoalveolar lavage; bid, twice daily; CrAg, cryptococcal antigen; CSF, cerebrospinal fluid; CT, computed tomography; FLC, fluconazole; ID, infectious diseases; IV, intravenous; micro, microbiology; L-AmB, liposomal amphotericin B; LP, lumbar puncture; MPA, mycophenolate acid; OP, opening pressure; PO, per os; pred, prednisone; prelim, preliminary; *S lugdunensis, Staphylococcus lugdunensis; S agalactiae, Streptococcus agalactiae*; tacro, tacrolimus (with target dose range in µg/L).

A 68-y-old man with end-stage interstitial lung disease underwent an elective double-lung transplant (Figure 3). Immunosuppression was tacrolimus (target trough 10–15 µg/L), azathioprine 100 mg/d later switched to mycophenolate acid (MPA) 720 mg 2× per day, and pulse methylprednisolone induction with prednisone taper.

Donor bronchoalveolar lavage (BAL) culture was initially positive for *Staphylococcus lugdunensis* and *Streptococcus agalactiae*, managed with 2-wk antibiotics. He progressed well posttransplant and was transferred to the ward.

Donor BAL culture resulted in *C neoformans* (pretransplant recipient BAL cultures negative) 11 d posttransplant, and high-dose fluconazole was started. The recipient’s serum CrAg was positive (titer 1:32). He was treated for donor-derived cryptococcosis using an AMBITION regimen. MPA was reduced to 540 mg 2× per day.

MRI brain was normal. LP 5 d after antifungal initiation showed clear and colorless CSF: opening pressure unavailable, WBC count 0 × 10^6^/L, glucose 4.5 mmol/L, protein 0.2 g/L, negative CrAg by latex agglutination, and negative bacterial and fungal culture and PCR.

Posttransplant day 19, he was clinically stabilized and discharged home to complete 2-wk oral backbone of the AMBITION induction protocol (flucytosine and high-dose fluconazole). He presented to the hospital 3-wk postdischarge with anorexia, malaise, and progressive right-sided pleural effusion confirmed on CT chest. MPA was held for leukopenia.

Empiric piperacillin-tazobactam was administered. Pleural fluid analysis suggested exudative effusion: pH 7.6, WBC count 464 × 10^6^/L (59% macrophages, 22% neutrophils, and 19% lymphocytes), red blood cell count 22 000 × 10^6^/L, lactate dehydrogenase 1469 U/L (serum 265 U/L), protein 25 g/L (serum 57 g/L), and negative cytology. Pleural fluid cultures were negative for bacteria, mycobacteria, and fungi. Repeat serum CrAg was negative. Although *Cryptococcus*-related effusion was possible, management was nonetheless unchanged with ongoing antifungal therapy, and pleural effusion resolved postthoracentesis.

With improved clinical and biochemical parameters, he was discharged home with a step down to consolidation, then he planned 1-y maintenance with fluconazole monotherapy. He resumed reduced-dose MPA 180 mg 2× per day 3 wk later and remained clinically well at a 5-mo follow-up.

## DISCUSSION

These first 2 reported cases of cryptococcosis in SOT recipients treated with a AMBITION single-dose l-AmB induction regimen demonstrated clinical efficacy and satisfactory adverse effect profiles. Future cohort studies with longer follow-ups should examine this treatment regimen in SOT recipients.

The AMBITION regimen was well tolerated by both patients described. Despite lower nephrotoxicity than AmBd, AKI occurs in more than one-third of patients receiving l-AmB, more so with higher doses or concomitant immunosuppressive or nephrotoxic agents. Calcineurin inhibitor (CNI) levels may be increased when triazoles are used to treat cryptococcosis, with supratherapeutic levels compounding AKI risk.^9^ Although patient 1 experienced AKI, multiple risk factors included preexisting kidney dysfunction and pre–renal hypovolemic insults. Notably, AKI in kidney transplantation raises the possibility of allograft rejection after immunosuppression reduction to manage infections, thus highlighting the challenges of balancing immunosuppression in SOT recipients with cryptococcosis. Indeed, a 30-d graft loss was 18% among kidney transplant recipients after cryptococcosis treatment with AmBd.^4^ Reassuringly, both cases returned to baseline kidney function, and there were no other adverse effects of AmB, including electrolyte derangements, cytopenias, infusion reaction, or transaminitis. Although patient 1 developed a headache, which may occur with fluconazole, this was likely related to concurrent CNS cryptococcosis with elevated intracranial pressure as it subsequently resolved with LP. No other adverse effects attributable to the AMBITION regimen occurred in either patient during follow-up including flucytosine-associated hepatotoxicity or bone marrow suppression.

These cases reinforce a need for studies in SOT recipients, a high-risk population with increased mortality, and unique considerations for cryptococcosis management.^2^ Worse outcomes may follow an irreversible immunocompromised state, delayed diagnosis and treatment, drug interactions, or allograft dysfunction. Nonspecific or atypical clinical presentations contribute to delayed diagnosis in SOT recipients. Patient 1 presented with cryptococcuria, a rare presentation (incidence 0.56/10 000 patients), and developed possible prostatic abscesses, with only ~70 cases of prostatic involvement reported since the 1940s.^3,10,11^ Diagnosis is further complicated by false-negative tests. CrAg may be negative with low fungal burden and prozone phenomenon from high antigen titers, and, rarely, hypocapsular or acapsular *Cryptococcus* strains.^12,13^ CSF PCR as an alternative to gold-standard fungal culture may be falsely negative with low fungal burden or antifungal exposure.^2^ Both patients received at least 1 antifungal dose before testing and were on tacrolimus, a CNI with in vitro antifungal activity, which is associated with a lower risk of disseminated disease and mortality because of *Cryptococcus*.^14^ Adverse effects of antifungals may be compounded by overlapping risk profiles with immunosuppressive agents, including nephrotoxicity with CNIs, or anemia with antimetabolites and may result in treatment interruptions.^5^

There are healthcare implications for adopting the AMBITION regimen in SOT recipients. A shorter duration of l-AmB and reduced adverse effects may lead to fewer intravenous administrations, shorter hospitalization, and decreased adverse event monitoring, with cost savings throughout.^6,15^ The AMBITION regimen was cost-effective among PLWH in sub-Saharan African countries, with a low incremental cost-effectiveness ratio of $80 ($15–275) (USD) per life-year saved.^16^ A substudy of the AMBITION trial interviewed patients, healthcare providers, and researchers, who expressed a preference for the AMBITION regimen.^17^ Studies exploring the pragmatism of AMBITION regimen are needed in high-income settings where the standard regimen is 2-wk l-AmB 3–4 mg/kg/d with flucytosine 100 mg/kg/d, *Cryptococcus*-associated mortality is lower, and adverse effects may be less tolerated.

These cases highlight nuanced clinical presentations, diagnostic challenges, and treatment considerations for SOT recipients with cryptococcosis successfully treated with an AMBITION single-dose l-AmB induction regimen. Additional studies are needed to evaluate the efficacy and safety of this regimen in SOT recipients, identify treatment candidates, and explore logistical issues for clinical deployment.

## ACKNOWLEDGMENTS

The authors thank the patients for their permission to publish this case report. They also thank Shoko Mineki for her help in preparing a visual graphic presentation of patient cases in Figures 1 and 3. They also thank the colleagues from the Toronto Lung Transplant Program, Department of Abdominal Surgery and Divisions of Transplant Infectious Diseases and Transplant Nephrology at University Health Network, Toronto, Ontario, Canada, for their useful suggestions and clinical contribution related to the patients’ care.
